# 

**Published:** 1867-02

**Authors:** 


### THE CHICAGO MEDICAL JOURNAL.

**E. L. HOLMES, M.D., H. M. LYMAN, M.D., R. M. LACKEY, M.D., Editors.**

All letters for the JOURNAL should be addressed to R. M. LACKEY, M.D., P.O. Box 2175, Chicago, Ill.

**TERMS---$2 PER ANNUM, IN ADVANCE.**

$3 if not paid before 1st of July in each year. Single copies, 25 cents.

